# Real‐World Treatment Patterns and Survival Outcomes in Metastatic Triple Negative Breast Cancer: Immunotherapy‐ Versus Anti‐Angiogenic Therapy‐Combined‐With‐Chemotherapy

**DOI:** 10.1002/cam4.71164

**Published:** 2025-09-16

**Authors:** Yingzhe Wang, Song Wu, Jianbin Li, Yang Yuan, Li Bian, Shaohua Zhang, Tao Wang, Zefei Jiang

**Affiliations:** ^1^ Department of Oncology The Fifth Medical Center of Chinese PLA General Hospital Beijing China; ^2^ Medical School of Chinese PLA Beijing China; ^3^ Department of Medical Molecular Biology Beijing Institute of Biotechnology, Academy of Military Medical Sciences Beijing China

**Keywords:** anti‐angiogenic therapy, chemotherapy, immunotherapy, metastatic triple‐negative breast cancer

## Abstract

**Purpose:**

There is limited clinical evidence comparing different chemotherapy‐based combination therapies. This study aimed to evaluate and compare the efficacy and safety of chemotherapy combined with immunotherapy versus chemotherapy combined with anti‐angiogenic therapy in the treatment of metastatic triple‐negative breast cancer (TNBC).

**Methods:**

This study included patients with metastatic TNBC who received either anti‐PD‐1 monoclonal antibody or bevacizumab in combination with chemotherapy. The primary endpoint was progression‐free survival (PFS); the secondary endpoints included overall response rate (ORR), clinical benefit rate (CBR), and safety.

**Results:**

Between October 2018 and June 2024, 130 eligible patients were enrolled. Of these, 60 patients received chemotherapy combined with anti‐PD‐1 monoclonal antibody, and 70 patients received chemotherapy combined with bevacizumab. The median PFS was 5.9 months (95% CI: 4.3–8.7) in the immunotherapy group, compared to 3.0 months (95% CI: 2.2–4.7) in the bevacizumab group (hazard ratio [HR] = 0.42, 95% confidence interval [CI] 0.28–0.62, *p* < 0.0001). The ORR rates were 55% in the immunotherapy group and 27.1% in the bevacizumab group (*p* = 0.001). The CBR rates were 43.3% and 22.9%, respectively (*p* = 0.013). The overall incidence of adverse events was comparable between the two groups.

**Conclusion:**

In the treatment of metastatic TNBC, chemotherapy combined with immunotherapy offers significant survival advantages over chemotherapy combined with bevacizumab.

## 1 Introduction

Breast cancer remains the leading malignant tumor threatening women's health today [1]. Different molecular subtypes of breast cancer exhibit distinct biological characteristics. Triple‐negative breast cancer (TNBC), which accounts for approximately 15%–20% of all breast cancer cases, is associated with a poor prognosis. Approximately 33.9% of patients with early‐stage TNBC will experience tumor recurrence or metastasis during the course of the disease, a rate that is notably higher than that observed in other molecular subtypes [2, 3].

Currently, both international and domestic authoritative guidelines, as well as expert consensus, recommend chemotherapy or chemotherapy in combination with other targeted therapies, such as immunotherapy or anti‐angiogenic agents, as first‐line treatment options for advanced TNBC [4, 5, 6, 7, 8]. Based on the findings of several recent clinical studies [9, 10, 11, 12, 13, 14, 15], the combination of chemotherapy with immunotherapy or anti‐angiogenic therapy has demonstrated superior efficacy compared to chemotherapy alone. However, within the context of current clinical practice, there is limited clinical evidence directly comparing the efficacy and safety of these two treatment regimens. This study aims to evaluate the efficacy and safety of chemotherapy combined with immunotherapy versus chemotherapy combined with anti‐angiogenic therapy in the treatment of metastatic TNBC.

## 2 Materials and Methods

### 2.1 Study Design and Population

All patients diagnosed with metastatic TNBC at our institution between October 2018 and June 2024 were enrolled in this study. Diagnosis was based on pathological confirmation, defined as negative or nearly negative estrogen receptor (ER) and progesterone receptor (PR) expression (1%–10%) and negative or low expression of human epidermal growth factor receptor 2 (HER2). Patients received chemotherapy in combination with either an anti‐PD‐1 monoclonal antibody (mAb) or bevacizumab. The most recent follow‐up date was September 1, 2024.

Eligibility criteria included being at least 18 years old with pathologically confirmed TNBC. Recurrences were identified through biopsy or imaging studies. Pathological types included invasive carcinoma and non‐metaplastic carcinoma; tumors of mesenchymal origin were excluded. Treatment in the first to third lines for metastatic TNBC was permitted. Patients who received chemotherapy combined with bevacizumab or anti‐PD‐1 monoclonal antibodies (including pembrolizumab, toripalimab, sintilimab, and camrelizumab) were eligible for inclusion. Additionally, patients with a history of hormone receptor‐positive expression were allowed to receive first‐line endocrine therapy. An Eastern Cooperative Oncology Group (ECOG) performance status score of 0–2 was required, and at least one measurable lesion, as assessed by the investigator, was necessary for eligibility.

### 2.2 Assessments

The efficacy of treatment in this study was assessed using the Response Evaluation Criteria in Solid Tumors (RECIST) version 1.1. Efficacy evaluations were conducted via computed tomography (CT) or magnetic resonance imaging (MRI) after every two cycles (6–8 weeks) during the treatment period. Secondary and additional endpoints included the investigator‐confirmed objective response rate (ORR), defined as the proportion of patients achieving a complete or partial response among all treated patients, and the clinical benefit rate (CBR), defined as the proportion of patients achieving a complete response, partial response, or stable disease lasting more than six months after treatment. To ensure consistency and minimize potential assessment bias, all imaging evaluations were reviewed by the same team of oncologists at our institution.

All adverse events (AEs) were recorded throughout the study. Treatment‐emergent adverse events were graded according to the Common Terminology Criteria for Adverse Events (version 5.0) and classified according to the Medical Dictionary for Regulatory Activities (version 27.0) [16].

### 2.3 Statistical Analysis

The primary endpoint of the study was progression‐free survival (PFS), defined as the time from the initiation of study treatment to either objective tumor progression or death, with patients without progression or death censored at the last follow‐up. Survival curves were generated using the Kaplan–Meier method, and group comparisons were conducted using the log‐rank test. Chi‐square tests were used to analyze differences in the ORR and CBR between treatment groups. Hazard ratios and their 95% confidence intervals were calculated using stratified Cox proportional hazards models in subgroup analyses. Safety analyses were performed to compare safety data between the two treatment regimens, excluding cases with missing adverse event records due to the retrospective nature of the data for patients who received at least one dose of the trial drug.

## 3 Oversight

This study is a clinical analysis based on retrospective medical records, and informed consent was not required for the inclusion of patients (CSCO BC RWS 2501). The study was approved by the Ethics Committee at our institution (KY‐2024‐11‐189‐1), and we are committed to adhering to relevant laws, regulations, and ethical standards to contribute to the improvement of medical quality and patient welfare.

## 4 Results

### 4.1 Patients

**FIGURE 1:**
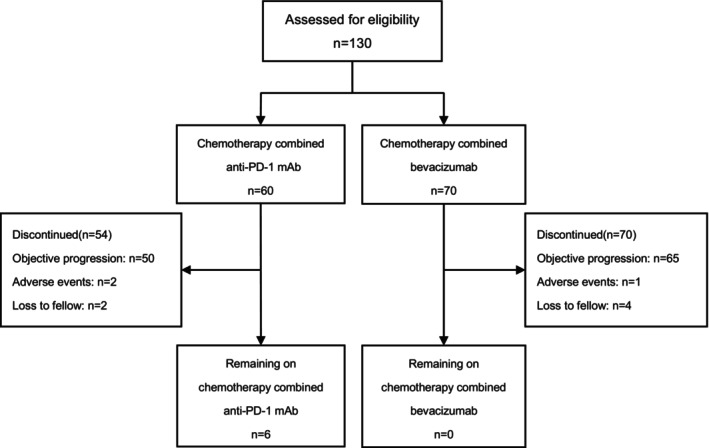
The study selection flow chart.

Between October 2018 and June 2024, 130 patients with metastatic TNBC were enrolled according to the inclusion and exclusion criteria of this study. The selection flowchart, shown in detail in Figure 1, outlines the patient enrollment process. The median age was 47.5 years (range: 22–69), and all participants were female. Of these, 60 received chemotherapy combined with an anti‐PD‐1 mAb, while 70 received chemotherapy with bevacizumab. The specific chemotherapy regimen was selected by the investigator based on the clinical context and included agents such as taxanes, gemcitabine, vinorelbine, and eribulin, etc. Notably, concurrent taxane use was higher in the anti‐PD‐1 mAb group (51.6%, 31/60) than in the bevacizumab group (27.1%, 19/70). In contrast, prior taxane exposure was comparable, with 85.0% (51/60) of patients in the anti‐PD‐1 mAb group and 92.9% (65/70) in the bevacizumab group having previously received taxane‐based therapy (*p* = 0.247). Visceral metastasis was present in 34 (56.7%) patients in the anti‐PD‐1 mAb group and 49 (70.0%) in the bevacizumab group. Patients received these therapies in first‐ to third‐line settings, with a minor difference in treatment lines between the groups, consistent with real‐world clinical practice. Baseline characteristics were generally balanced between the groups, except for the current line of therapy (Table 1).

**TABLE 1:** Baseline characteristics of patients.

Characteristics	Total (*n* = 130)	Chemotherapy plus anti‐PD‐1 mAb (*n* = 60)	Chemotherapy plus bevacizumab (*n* = 70)	*p*
Age, *n* (%)				
≤ 45	64 (49.2)	27 (45.0)	37 (52.9)	0.473
> 45	66 (50.8)	33 (55.0)	33 (47.1)	
Pathology, *n* (%)				
Invasive ductal carcinoma	116 (89.2)	53 (88.3)	63 (90)	0.983
Other	14 (10.8)	7 (11.7)	7 (10)	
TNM stage, *n* (%)				
1	15 (11.5)	6 (10.0)	9 (12.8)	0.664
2	64 (49.2)	32 (53.4)	30 (42.8)	
3	27 (20.8)	11 (18.3)	16 (22.8)	
4	24 (18.5)	11 (18.3)	13 (18.6)	
Treatment stage, *n* (%)				
De novo stage IV	24 (18.5)	11 (18.3)	13 (18.6)	0.998
DFI ≤ 12 m	37 (28.5)	17 (28.3)	20 (28.6)	
DFI > 12 m	69 (53.1)	32 (53.3)	37 (52.9)	
Previous taxanes treatment, *n* (%)				
No	14 (10.8)	9 (15.0)	5 (7.1)	0.247
Yes	116 (89.2)	51 (85.0)	65 (92.9)	
Current line of therapy, *n* (%)				
1	53 (40.8)	32 (53.3)	21 (30.0)	0.022
2	49 (37.7)	19 (31.7)	30 (42.9)	
3	28 (21.5)	9 (15.0)	19 (27.1)	
Visceral metastasis, *n* (%)				
No	47 (36.2)	26 (43.3)	21 (30.0)	0.163
Yes	82 (63.8)	34 (36.7)	49 (70.0)	

### 4.2 Efficacy

**FIGURE 2:**
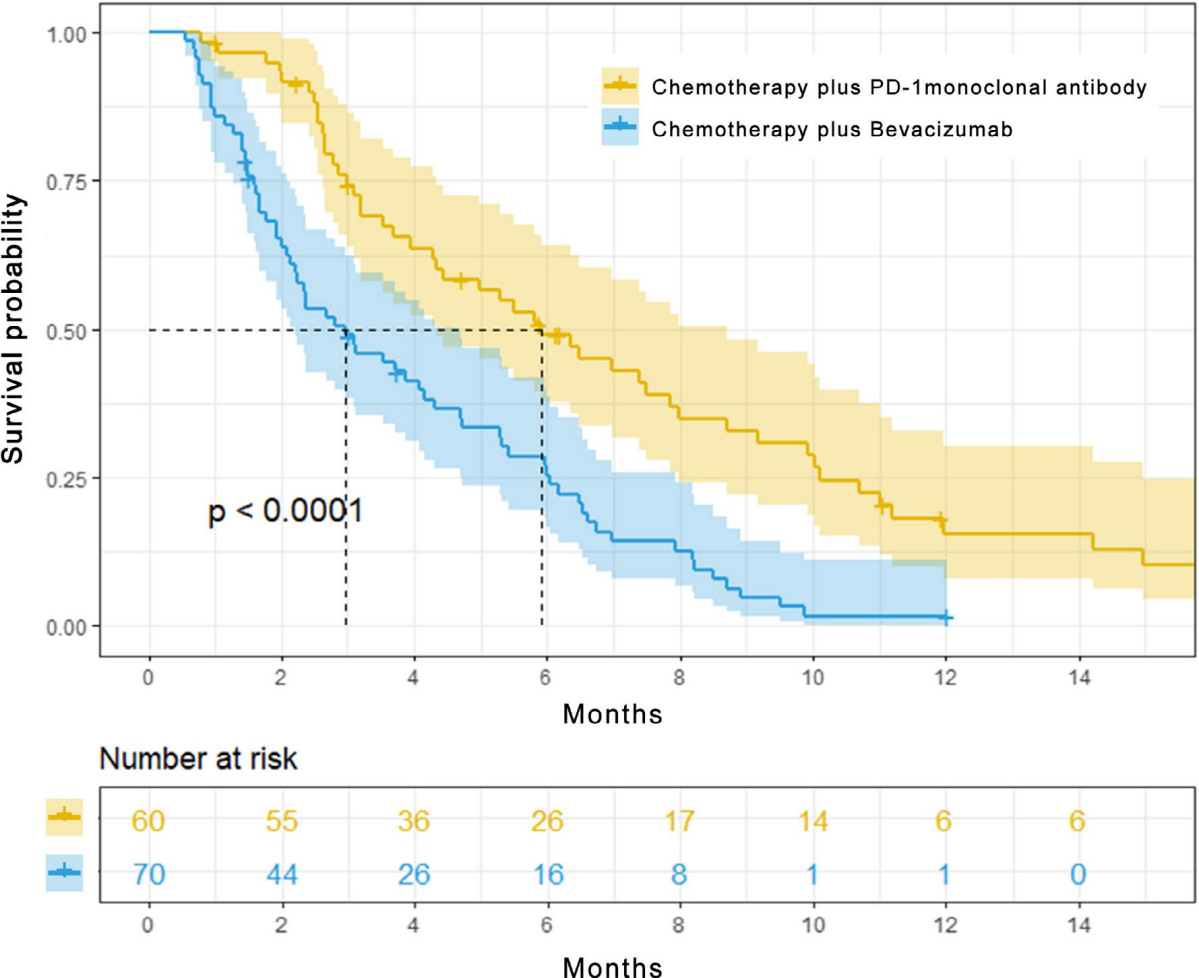
The PFS of chemotherapy combined with bevacizumab or anti‐PD‐1 mAb.

**FIGURE 3:**
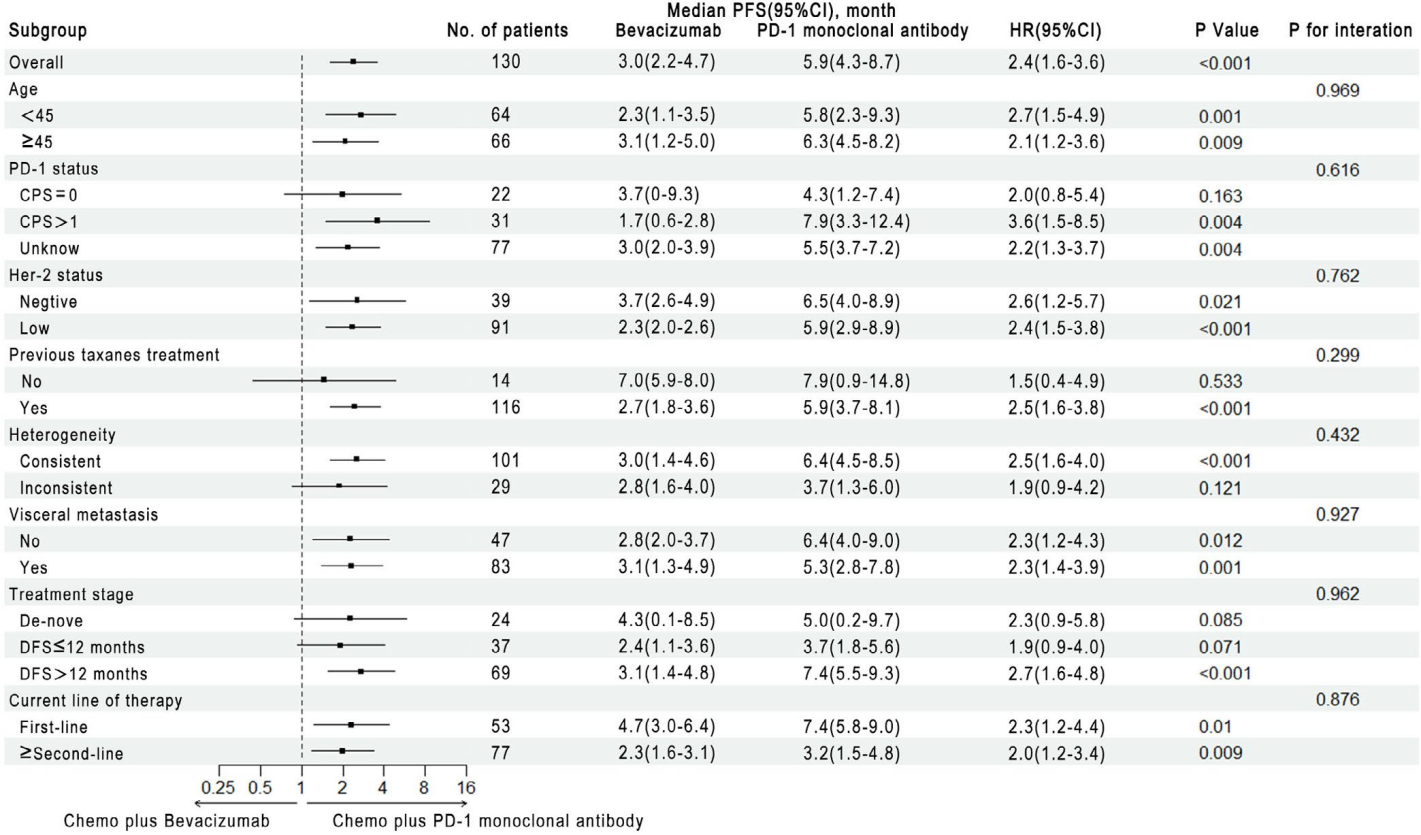
PFS in subgroups according to treatment at baseline.

At the time of the last follow‐up, 50 patients (83.3%) in the anti‐PD‐1 mAb group and 65 patients (92.9%) in the bevacizumab group reached the endpoint. The median PFS was 5.9 months (95% CI: 4.3–8.7) in the chemotherapy combined with anti‐PD‐1 mAb group and 3.0 months (95% CI: 2.2–4.7) in the chemotherapy combined with bevacizumab group (*p* < 0.0001). The hazard ratio for disease progression or death in the chemotherapy combined with anti‐PD‐1 mAb group was 0.42 (95% CI: 0.28–0.62; *p* < 0.0001) (Figure 2). Subgroup analyses based on various clinical factors—including age, PD‐1 expression status, HER2 expression status, prior treatment with or without taxanes, tumor heterogeneity, presence of visceral metastasis, treatment stage (initial diagnosis as stage IV, disease‐free survival (DFS) ≤ 12 months, DFS > 12 months), and current treatment lines—consistently demonstrated a superior benefit of chemotherapy combined with anti‐PD‐1 mAb over chemotherapy combined with bevacizumab in terms of PFS (Figure 3).

In all subgroups, including patients of all ages, those with low or absent HER2 expression, those with or without visceral metastasis, and those receiving first‐line or subsequent treatments, chemotherapy combined with anti‐PD‐1 mAb showed superior efficacy. In patients with a combined positive score (CPS) ≥ 1 and those without CPS testing, chemotherapy combined with anti‐PD‐1 mAb significantly outperformed chemotherapy combined with bevacizumab. Most patients in this study had previously received taxanes, and in this subgroup, the efficacy of chemotherapy combined with anti‐PD‐1 mAb was superior compared to the bevacizumab group. However, 14 patients in the study had not received prior taxane treatment. Of all patients included in this study, approximately 22% were not diagnosed with TNBC at the time of their initial diagnosis, highlighting the heterogeneity in the timing of tumor recurrence and metastasis. This finding is consistent with previous studies on tumor heterogeneity [17, 18, 19]. Considering tumor heterogeneity, the subgroup analysis suggests that the benefits of chemotherapy combined with anti‐PD‐1 mAb are particularly pronounced in patients who maintain triple‐negative status throughout recurrence and metastasis. Additionally, a trend indicates that patients with a DFS duration ≥ 12 months derive notable benefits from chemotherapy combined with anti‐PD‐1 mAb.

As shown in Table 2, the complete response (CR) rates in the anti‐PD‐1mAb group and bevacizumab group were 11.7% and 1.4%, respectively; the partial response (PR) rates were 43.3% and 25.7%, respectively; and the stable disease (SD) rates were 30.0% and 32.9%, respectively. The ORR and CBR were significantly higher in the anti‐PD‐1mAb group compared with the bevacizumab group. The ORR was 55.0% versus 27.1% (*p* = 0.001), and the CBR was 43.3% vs. 22.9% (*p* = 0.013).

**TABLE 2:** The efficacy of chemotherapy combined with bevacizumab or anti‐PD‐1 mAb.

Efficacy	Chemotherapy plus anti‐PD‐1 mAb, *n* (%)	Chemotherapy plus bevacizumab, *n* (%)	*p*
CR	7 (11.7)	1 (1.4)	
PR	26 (43.3)	18 (25.7)	
SD	18 (30.0)	23 (32.9)	
PD	9 (15.0)	28 (40.0)	
Objective response rate (CR + PR)	33 (55.0)	19 (27.1)	0.001
Clinical benefit rate (CR + PR + SD > 6 months)	26 (43.3)	16 (22.9)	0.013

### 4.3 Safety and Adverse Events

The safety assessment population included 119 patients who received at least one therapeutic dose, with 56 patients in the chemotherapy combined with anti‐PD‐1 mAb group and 63 patients in the chemotherapy combined with bevacizumab group. The overall incidence of AEs of any grade was comparable between the two groups, at 96.5% (54/56) for the anti‐PD‐1 group and 95.3% (60/63) for the bevacizumab group. The most common treatment‐related AEs of any grade were anemia (39.3% vs. 27.0%), leukopenia (58.9% vs. 47.6%), nausea and vomiting (12.8% vs. 11.8%), abnormal liver function (42.9% vs. 25.4%), and peripheral neuritis or hand‐foot syndrome (50.0% vs. 42.9%). Grade 3–4 adverse reactions occurred in 17.9% (10/56) of patients in the anti‐PD‐1 mAb group and 9.5% (6/63) in the bevacizumab group, with hematologic toxicities—particularly leukopenia and thrombocytopenia—being the most common severe events.

In terms of treatment discontinuation due to AEs, two patients (3.3%) in the anti‐PD‐1 mAb group discontinued therapy: one due to immune‐related hepatitis and another due to immune‐related encephalitis. In the bevacizumab group, one patient (1.4%) discontinued treatment due to hemorrhagic complications. Among the patients who continued receiving anti‐PD‐1 immunotherapy (*n* = 58), immune‐related adverse events (irAEs) were observed in several cases. These included endocrine disorders in nine patients (seven cases of thyroid dysfunction and two cases of hypothalamic–pituitary–adrenal axis suppression) and cardiac arrhythmias in two patients. Importantly, no unexpected safety signals or new immune‐related toxicities were detected during the study (Table 3).

**TABLE 3:** The safety of chemotherapy combined with bevacizumab or anti‐PD‐1 mAb.

Adverse event	Chemotherapy plus anti‐PD‐1 mAb (*N* = 56)		Chemotherapy plus bevacizumab (*N* = 63)	
	Any grade	Grade 3–4	Any grade	Grade 3–4
	Number of patients (percent)			
Any adverse event	54 (96.5)	10 (17.9)	60 (95.2)	6 (9.5)
Hematologic event				
Anemia	22 (39.3)	0	17 (27.0)	0
Leukopenia	33 (58.9)	8 (14.3)	30 (47.6)	5 (7.9)
Thrombocytopenia	4 (7.1)	3 (5.4)	2 (3.2)	0
Gastrointestinal event	5 (8.9)	0	7 (11.1)	0
Abnormal liver function	24 (42.9)	1 (1.8)	16 (25.4)	0
General disorders				
Asthenia and fatigue	10 (17.9)	0	1 (1.6)	0
Peripheral neuritis and hand‐foot reactions	28 (50.0)	1 (1.8)	27 (42.9)	1 (1.6)

## 5 Discussion

This study, grounded in our real‐world clinical practice, compared the efficacy and safety of chemotherapy combined with anti‐PD‐1 mAb to chemotherapy combined with bevacizumab in patients with metastatic TNBC. As is known, there is limited clinical evidence directly comparing the efficacy and safety of these two treatment regimens. Our findings raise several encouraging implications. First, patients in the anti‐PD‐1 mAb group had a longer PFS than that in the bevacizumab group, with improvements also observed in the ORR and CBR. Second, subgroup analyses consistently showed results similar to those of the overall study population. In addition, our study confirmed a comparable overall incidence of AEs in the anti‐PD‐1 mAb group compared with the bevacizumab group, except for AEs specifically related to immunotherapy or anti‐angiogenic therapy.

Our results suggest that chemotherapy combined with anti‐PD‐1 mAb significantly improved PFS compared to chemotherapy combined with bevacizumab in patients with metastatic TNBC. This improvement also reflected in the ORR and CBR. A wealth of research indicates that the combination of chemotherapy with anti‐PD‐1 mAb or bevacizumab yields significant results in the first‐line treatment of advanced breast cancer, providing a strong foundation for further investigation. However, in real‐world clinical practice, not all metastatic TNBC patients receive chemotherapy‐based combination therapy as first‐line treatment due to differences in drug availability and physician treatment philosophies. Furthermore, previous clinical studies included patients with all molecular subtypes in first‐line treatment, rather than focusing exclusively on those with metastatic TNBC. Given this clinical research background and our real‐world clinical observations, we designed this study. The Keynote‐355 study indicates that in patients with metastatic TNBC and a composite positive score (CPS) of ≥ 10, the combination of chemotherapy and anti‐PD‐1 mAb significantly enhances PFS and overall survival (OS) compared to chemotherapy alone as first‐line treatment [9, 10]. Additionally, the Torchlight study shows that for metastatic TNBC patients with a CPS of ≥ 1, the combination of chemotherapy and anti‐PD‐1 mAb leads to an extension of PFS and OS, with 95% of participants receiving this treatment as their first‐line treatment [11, 12]. Results from the E2100, AVADO, and RIBBON‐1 studies suggest that a statistically significant improvement of PFS in combination with bevacizumab compared to chemotherapy alone, particularly within the triple‐negative subgroup [13, 14, 15]. However, OS analysis from those studies indicates that this combination does not lead to an improvement in overall survival. A review of the literature indicates that previous clinical trials evaluating the combination of chemotherapy and bevacizumab in metastatic breast cancer included patients with various molecular subtypes, with TNBC patients comprising approximately 20%–25% of the participants. Subgroup analyses have demonstrated that the combination of chemotherapy and bevacizumab provides significant benefit in PFS for TNBC patients. However, the lack of OS benefit in these studies can likely be attributed to the fact that patients with other molecular subtypes typically had access to more endocrine or targeted therapies [14, 15, 20, 21]. Prior studies suggest that combining chemotherapy with immunotherapy may provide clinical benefits for TNBC patients in second‐line and later treatment phases compared to chemotherapy alone [22, 23]. In the treatment of metastatic TNBC, the safety profile remained favorable for all patients involved. The AEs associated with immune checkpoint inhibitors observed in this study align with the known risks reported in previous clinical studies, with no new safety concerns identified [24]. These findings reinforce the clinical value of incorporating anti‐PD‐1 immune checkpoint blockade therapy into chemotherapy for metastatic TNBC.

As a real‐world study investigating the efficacy of chemotherapy combined with either anti‐PD‐1 mAb or bevacizumab for metastatic TNBC, this research has several limitations. These include its retrospective nature from a single‐center database, a small sample size, and the lack of randomization, which creates an inevitable risk of selection bias. The study is also limited by the non‐standardized chemotherapy regimens. In this real‐world setting, investigators selected chemotherapy based on clinical judgment and patient history. This variability is an inherent confounding factor that could influence the interpretation of both efficacy and toxicity outcomes. A further limitation relates to our subgroup analyses, where we acknowledge that confounding factors such as the line of therapy and the presence of visceral metastases could influence the results. Propensity score matching (PSM) was not utilized to adjust for these imbalances. This decision was made deliberately, as applying PSM to a limited sample size (*n* = 130) would carry a significant risk of reducing statistical power and could lead to overmatching. Instead, to address this, we conducted stratified univariable Cox analyses. These analyses consistently demonstrated a PFS benefit with immunotherapy across critical subgroups, supporting the primary analysis and showing no significant heterogeneity (all interaction *p* > 0.05).

Despite these methodological challenges, the findings remain robust, supporting the prioritization of immunotherapy‐based regimens for advanced metastatic triple‐negative breast cancer (TNBC). Future research will focus on several key areas: (1) evaluating the impact of treatment sequencing, particularly the timing of immunotherapy relative to anti‐angiogenic therapy, on overall survival; (2) identifying predictive biomarkers to optimize patient selection and personalize treatment strategies; and (3) characterizing the intrinsic heterogeneity within patient populations that drives differential responses to combination therapies. These priorities will inform the design of larger prospective trials and facilitate the development of refined algorithms and predictive models for long‐term outcomes in metastatic TNBC.

## 6 Conclusion

In the treatment of patients with metastatic triple‐negative breast cancer, chemotherapy combined with immunotherapy is the preferred option, offering significant survival benefits compared to chemotherapy combined with anti‐angiogenic therapy, while overall adverse reactions remain manageable. Therefore, chemotherapy combined with immunotherapy should be prioritized for the treatment of metastatic triple‐negative breast cancer.

## Author Contributions

**Yingzhe Wang:** data curation (equal), formal analysis (equal), project administration (equal), writing – original draft (lead). **Song Wu:** data curation (equal), formal analysis (equal), project administration (equal). **Jianbin Li:** funding acquisition (equal), writing – original draft (equal), project administration (equal), (corresponding author). **Yang Yuan:** data curation (equal), project administration (equal). **Li Bian:** data curation (equal), project administration (equal), writing – review and editing (equal). **Shaohua Zhang:** project administration (equal), writing – review and editing (equal). **Tao Wang:** project administration (equal), writing – review and editing (equal). **Zefei Jiang:** funding acquisition (lead), project administration (equal), writing – review and editing (equal) (corresponding author). All authors reviewed and approved the final manuscript. Both Zefei Jiang and Jianbin Li are corresponding authors.

## Ethics Statement

The study was approved by the Ethics Committee of the Fifth Medical Center of Chinese PLA General Hospital (approval number: KY‐2024‐11‐189‐1).

## Conflicts of Interest

The authors declare no conflicts of interest.

## Data Availability

The data that support the findings of this study are available on request from the corresponding author. The data are not publicly available due to privacy or ethical restrictions.
